# Glibenclamide Posttreatment Does Not Inhibit Levcromakalim Induced Headache in Healthy Participants: A Randomized Clinical Trial

**DOI:** 10.1007/s13311-023-01350-y

**Published:** 2023-02-10

**Authors:** Lili Kokoti, Mohammad Al-Mahdi Al-Karagholi, Cherie Amalie Waldorff Nielsen, Messoud Ashina

**Affiliations:** 1 Danish Headache Center, Department of Neurology, Rigshospitalet – Glostrup, Faculty of Health and Medical Sciences, University of Copenhagen, Valdemar Hansen Vej 5, 2600 Glostrup, Denmark; 2 Danish Headache Knowledge Center, Rigshospitalet – Glostrup, Glostrup, Denmark

**Keywords:** Humans, Migraine, Cromakalim, Adenosine triphosphate-sensitive potassium channel, K_ATP_-channel

## Abstract

Intravenous infusion of ATP-sensitive potassium channel (K_ATP_) opener levcromakalim causes headache in humans and implicates K_ATP_ channels in headache pathophysiology. Whether K_ATP_ channel blocker glibenclamide inhibits levcromakalim-induced headache has not yet been elucidated. We aimed to investigate the effect of posttreatment with glibenclamide on levcromakalim-induced headache in healthy participants. In a double blind, randomized, three-arm, placebo-controlled, crossover study, 20 healthy participants were randomized to receive 20 mL of levcromakalim (0.05 mg/min (50 mg/mL)) or 20 mL placebo (isotonic saline) intravenously over 20 min followed by oral administration of 10 mg glibenclamide or placebo. Fifteen participants completed all three study days. The primary endpoint was the difference in incidence of headache (0–12 h) between glibenclamide and placebo. More participants developed headache on levcromakalim-placebo day (15/15, 100%) (*P* = *0.013*) and levcromakalim-glibenclamide day (13/15, 86%) compared to placebo-placebo day (7/15, 46%) (*P* = *0.041*). We found no difference in headache incidence between levcromakalim-placebo day and levcromakalim-glibenclamide day (*P* = *0.479*). The AUC_0–12 h_ for headache intensity was significantly larger in levcromakalim-placebo day and levcromakalim-glibenclamide day compared to placebo-placebo day (106.3 ± 215.8) (*P* < *0.01*). There was no difference in the AUC_0–12 h_ for headache intensity between the levcromakalim-placebo day (494 ± 336.6) and the levcromakalim-glibenclamide day (417 ± 371.6) (*P* = *0.836*). We conclude that non-specific K_ATP_ channel inhibitor glibenclamide did not attenuate levcromakalim-induced headache in healthy volunteers. Future studies should clarify the involvement of the distinct isoforms of sulfonylurea receptor subunits of K_ATP_ channels in the pathogenesis of headache and migraine.

**Supplementary Information:**

The online version contains supplementary material available at 10.1007/s13311-023-01350-y.

## Introduction

Experimental studies in humans suggest that ATP-sensitive potassium (K_ATP_) channels are involved in the signaling cascades underlying headache and migraine [1]. K_ATP_ channels are activated by various K^+^ channel openers including diazoxide, levcromakalim, and pinacidil and are inhibited by oral hypoglycemic drugs such as glibenclamide and tolbutamide [2]. These channels are expressed at several levels of the trigeminal pain pathway, including vascular smooth muscle cells (VSMCs), perivascular fibers, the trigeminal ganglion (TG) and the trigeminal nucleus caudalis (TNC) [3, 4]. Direct activation of K_ATP_ channels by levcromakalim infusion induced headache in healthy participants and migraine attacks in migraine patients [5, 6]. Moreover, endogenous intermediate molecules implicated in headache and migraine depend on activation of K_ATP_ channels [1, 7–9].

Preclinical studies reported that K_ATP_ channel inhibitor glibenclamide completely blocks trigeminal pain transmission in models of provoked migraine-like pain [10, 11]. Given that all available drugs in current clinical practice are only partially effective in the abortive treatment of migraine, the K_ATP_ channel may be a promising new downstream target for the treatment of migraine. We hypothesized that glibenclamide posttreatment would attenuate levcromakalim-induced headache. To test our hypothesis, we conducted a double blind, randomized, three-arm, placebo-controlled study in healthy participants.

## Material and Methods

### Participants

Twenty healthy participants were recruited via the Danish recruitment website www.forsoegsperson.dk, from June 2020 until March 2021. We aimed to evaluate the physiological effect of glibenclamide after levcromakalim in healthy participants as this would provide evidence for further investigation of it in migraine patients. Eligible participants were invited at the Danish Headache Center for detailed screening and medical examination. Written informed consent was obtained from all participants prior to enrolment at the study. Participants were informed that levcromakalim might induce headache, but its timing and characteristics were not discussed. Participants were also informed that glibenclamide is an anti-diabetic medication, and it is unknown whether it can affect levcromakalim-induced headache. The study was approved by the Regional Health Research Ethics Committee of the Capital Region (H-18052188) and the Danish Data Protection Agency. It was conducted according to the Declaration of Helsinki of 1964, with later revisions. The study was registered at ClinicalTrials.gov (NCT03886922) as part of a larger study protocol. The main study, as described in the study protocol and in the ClinicalTrials.gov, comprises two experimental studies: (i) a study investigating the effect of glibenclamide as pre-treatment on levcromakalim-induced vascular changes and headache in healthy volunteers (results reported elsewhere) and (ii) a study investigating the effect of glibenclamide as posttreatment on levcromakalim-induced vascular changes (results reported elsewhere) and headache in healthy volunteers (the present study).

### Inclusion/Exclusion Criteria

Participants were eligible for the study if they (1) were aged between 18 and 60 years old, and (2) weighted from 50 to 100 kg. All female participants used a sufficient contraceptive method (contraceptive pill or intrauterine device/system). Exclusion criteria were (1) anamnestic and/or clinical signs of serious somatic or psychiatric disease, (2) diagnosis of primary or secondary headache disorder according to ICHD-3 (except tension type headache less than 5 days per month) [12], (3) first-degree relative suffering from migraine or diabetes mellitus, (4) intake of daily medication (except contraceptives), and (5) pregnant and breastfeeding women. One of the authors (LK) evaluated eligibility, obtained informed consent, and enrolled the participants. Experiments were carried out at the Danish Headache Center, Department of Neurology, Rigshospitalet Glostrup, from 11 June 2020 to 15 March 2021.

### Experimental Design and Randomization

**Fig. 1:**
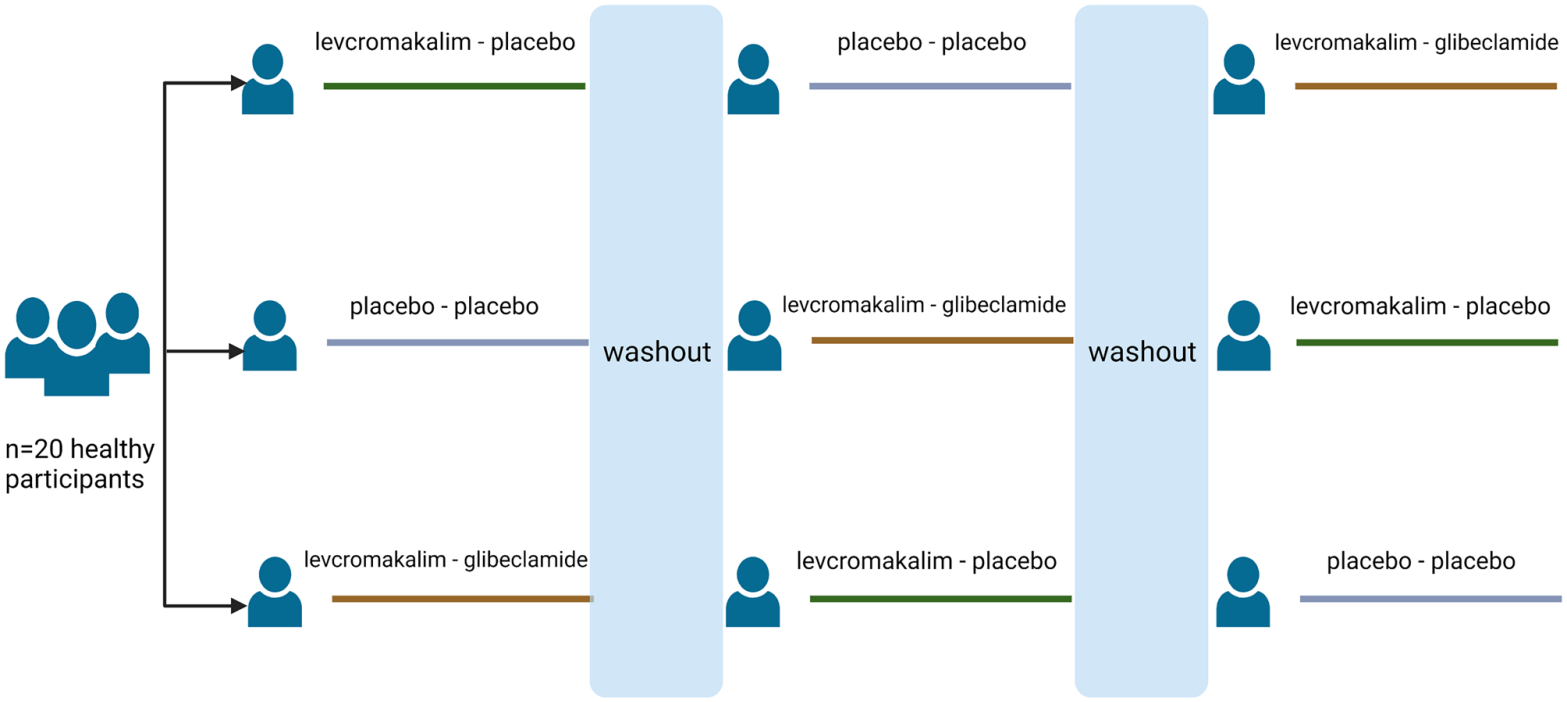
Experimental design. Healthy participants (*n* = 20) were randomly allocated in a double-blind crossover design, to receive levcromakalim followed by placebo (*n* = 7), levcromakalim followed by glibenclamide (*n* = 7), and placebo followed by placebo (*n* = 6) on three separate study days. There was a washout period of at least one week between each study day

We conducted a double-blind, placebo-controlled, three-arm, crossover study. All enrolled participants came at the clinic on three different study days separated by at least 1 week where they were randomly allocated without restrictions to receive a continuous intravenous infusion of 20 mL levcromakalim (0.05 mg/min (50 mg/mL) (Sigma-Aldrich, Darmstadt, Germany)) or 20 mL placebo (isotonic saline) over 20 min, followed by oral administration of glibenclamide (10 mg oral tablet of Hexaglucon, Sandoz) or placebo (multivitamin pill) 80 min afterwards. The three study days were the following: infusion of levcromakalim followed by oral placebo (levcromakalim-placebo day), levcromakalim infusion followed by oral glibenclamide (levcromakalim-glibenclamide day), and infusion of placebo followed by oral placebo (placebo-placebo day) (Fig. 1). Study drugs were prepared by the Capital Region Central Pharmacy. The randomization code remained in the hospital during the study and was not accessible to the investigators until the study was completed and the data were analyzed.

**Fig. 2:**
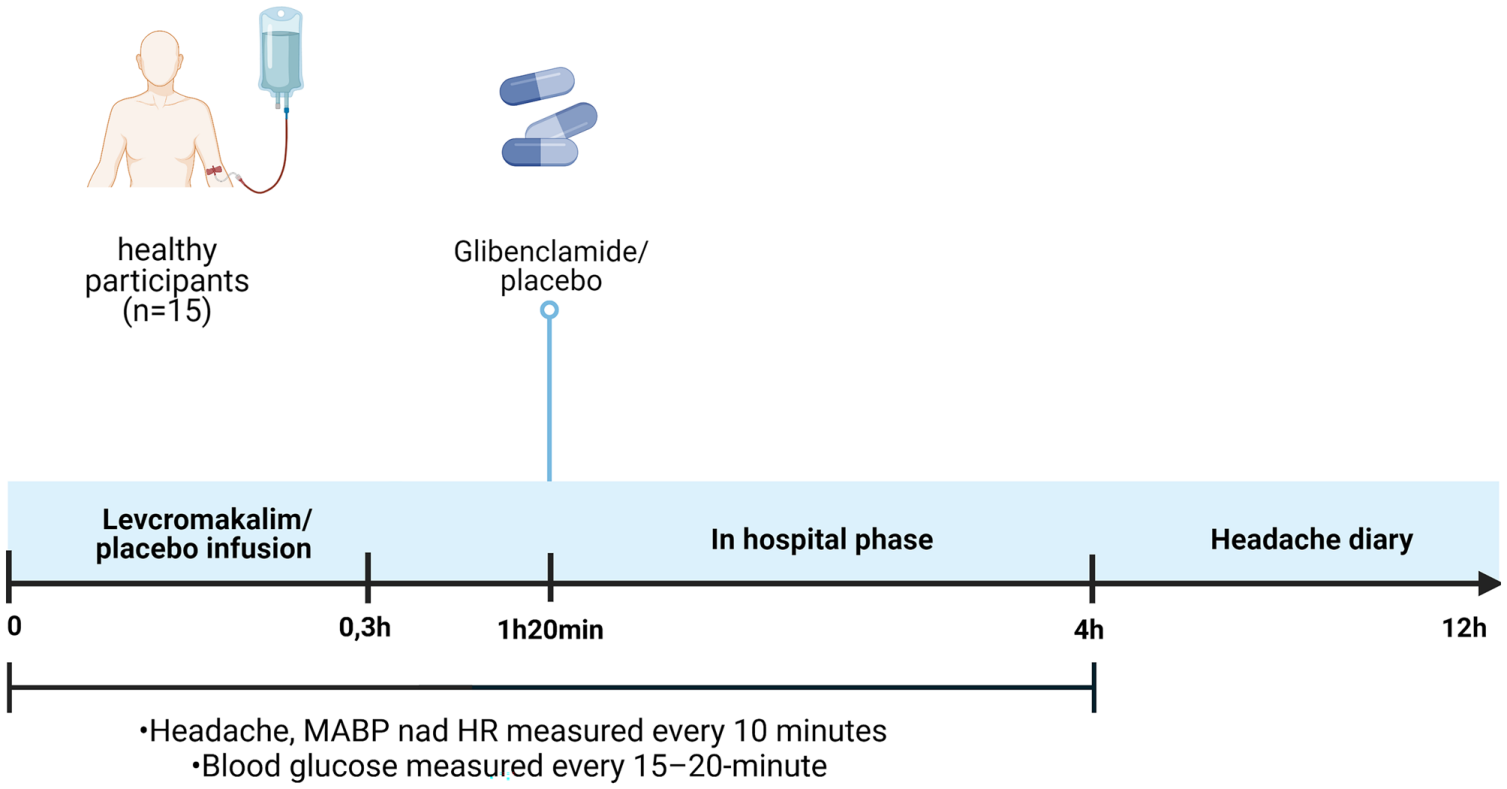
Experimental timeline. There was a 240-min in-hospital phase of the experiment and 8 h of outpatient headache diary. *MABP* mean arterial blood pressure, *HR* heart rate

Participants arrived at the clinic non-fasting, at the same time on each study day (± 2 h). Participants were at least 48 h headache-free and were not allowed to consume coffee, tea, cocoa, alcohol, and tobacco 12 h prior to study onset. All female participants were tested for pregnancy on all study days. A venous catheter (BD VenflonVR, Franklin Lakes, NJ) was inserted into the left and right antecubital vein for drug (levcromakalim/placebo) and 20% glucose infusion. The infusion started using a time and volume-controlled infusion pump. Mean arterial blood pressure (MABP) and heart rate (HR) were continuously monitored and recorded at baseline (T0) and every 10 min after the start of the infusion (Fig. 2).

### Headache and Accompanying Symptoms

A blinded investigator (LK and CWN) obtained data on headache intensity and characteristics at baseline (T0) and every 10 min after the start of the infusion (T0) until T240 minutes using a standardized questionnaire. Headache intensity was recorded on a numerical rating scale (NRS) from 0–10 (0, no headache; 1, very mild headache [including a pressing or throbbing feeling]; 10, worst imaginable headache). Headache characteristics including localization, pain quality, aggravation by physical activity, and associated symptoms (nausea, photophobia, and phonophobia) were also recorded. After being discharged from the hospital, participants were asked to complete a headache diary every hour until 12 h following the infusion. Apart from headache intensity and characteristics, the diary recorded uptake of analgesics and possible adverse events (AE). Participants were allowed to take medication if the headache became intolerable. The standard rescue medication provided on site was 1000 mg paracetamol and 400 mg ibuprofen.

### Blood Glucose Levels

Blood glucose levels were monitored during the experiment. Thirty minutes after posttreatment with glibenclamide or placebo (T110) and when initial fasting glycaemia had declined by 10%, blood glucose concentrations were clamped around 4–7 mmol/L by 20% glucose infusion. Infusion rates necessary to maintain blood glucose after drug intake were registered throughout the experiment. Twelve blood samples were obtained for the determination of glucose during the experimental period. Blood samples were drawn prior to glibenclamide/placebo administration and then with 15–20-min intervals starting at T110. The venous blood samples were drawn from the intravenous catheter using a blood gas aspirator (Radiometer, SafePICO, self-filling blood gas syringe), and the blood glucose concentrations were determined with a blood gas analyzer (Radiometer, ABL90 FLEX). After finishing the measurements (T240), participants were provided with a standardized meal rich in carbohydrates which they had to consume before getting discharged.

### Data Analysis and Statistical Analysis

All absolute values are presented as mean ± standard deviation (SD). Headache intensity and duration scores are presented as median (range). Calculation of sample size was based on detection of a difference between treatments in headache incidence of 80% on the placebo day and 20% on the glibenclamide day at a 5% significance level with 80% power. R statistical software (version 4.2.1) was used to calculate the sample size, using the “power.prop.test” function and “stats” package.

Sample size was calculated at 10 participants, and 15 participants were included to ensure power. The primary endpoint was difference in incidence of headache (0–12 h) between the three experimental days: levcromakalim-placebo day, levcromakalim-glibenclamide, and placebo-placebo day. The secondary endpoints were a difference in AUC for headache intensity scores (0–12 h) and HR and MABP from baseline to the last measurement between the three study days. Baseline was defined as T0 before the start of infusion.

Incidence of headache was analyzed as categorical paired data using McNemar’s test. Area under the curve (AUC) was calculated according to the trapezium rule to obtain summary measures and to analyze the differences in response between the three study days. AUC was compared between study drugs using the Wilcoxon signed-rank test.

Statistical analysis and graphs were performed using GraphPad Prism 8.3.0 (San Diego, CA, USA). Level of significance at five percent (*P* < *0.05*, two-tailed) was accepted for all tests. We did not adjust for multiple comparisons, as our primary endpoints, hypotheses, and statistical tests were not many, and they all were predefined and clearly stated in study protocol.

## Results

### Participant Characteristics

**Fig. 3:**
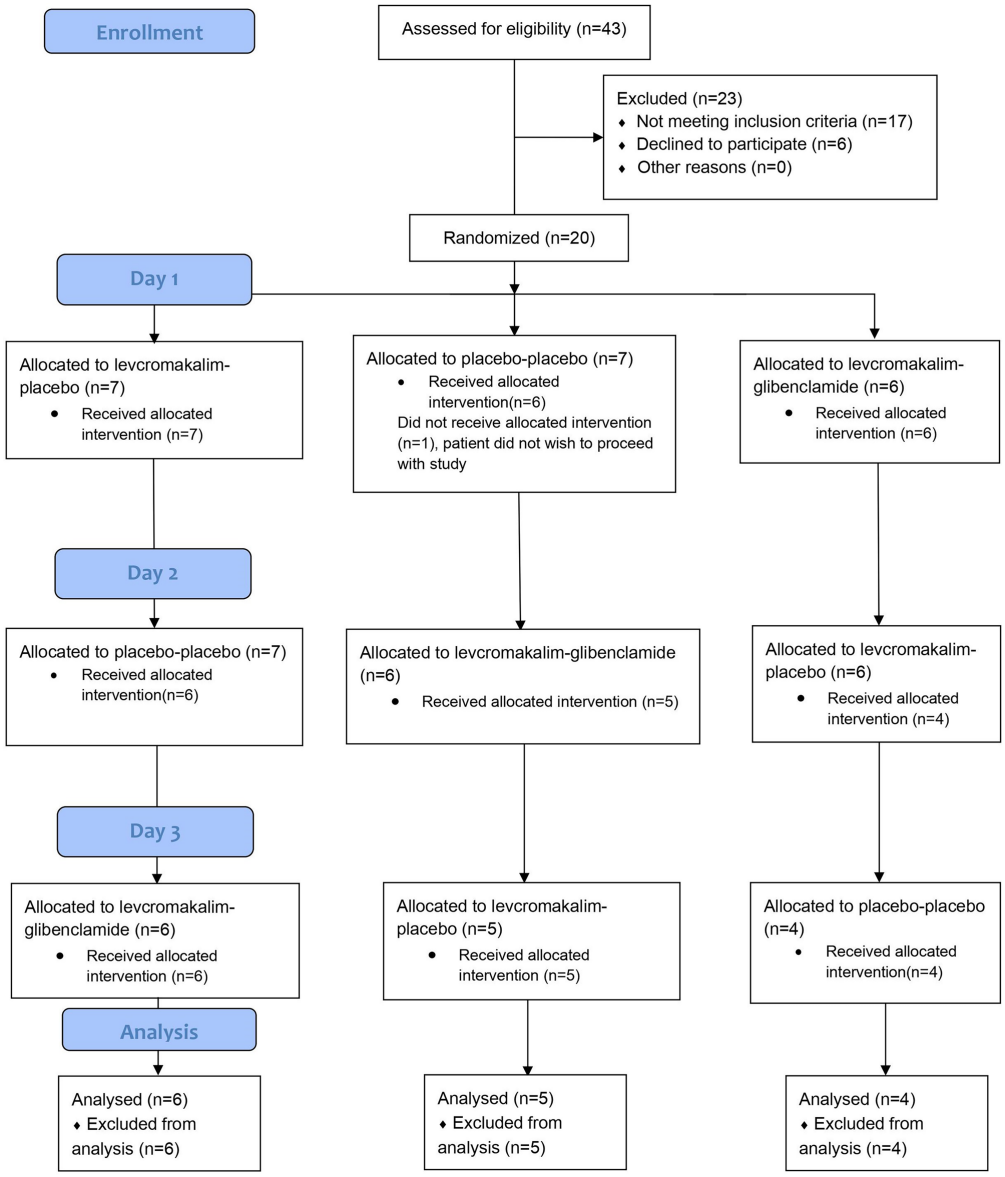
CONSORT flow chart of the study

Twenty healthy participants were enrolled in the study, and fifteen (75%) completed all three study days and were included in the final analysis (9 women and 6 men) (Fig. 3). Mean age of participants was 24.4 (range 22–27) and mean weight 65.2 kg (range 55–88). At baseline, there were no differences for any assessed variable.

### Headache

**Fig. 4:**
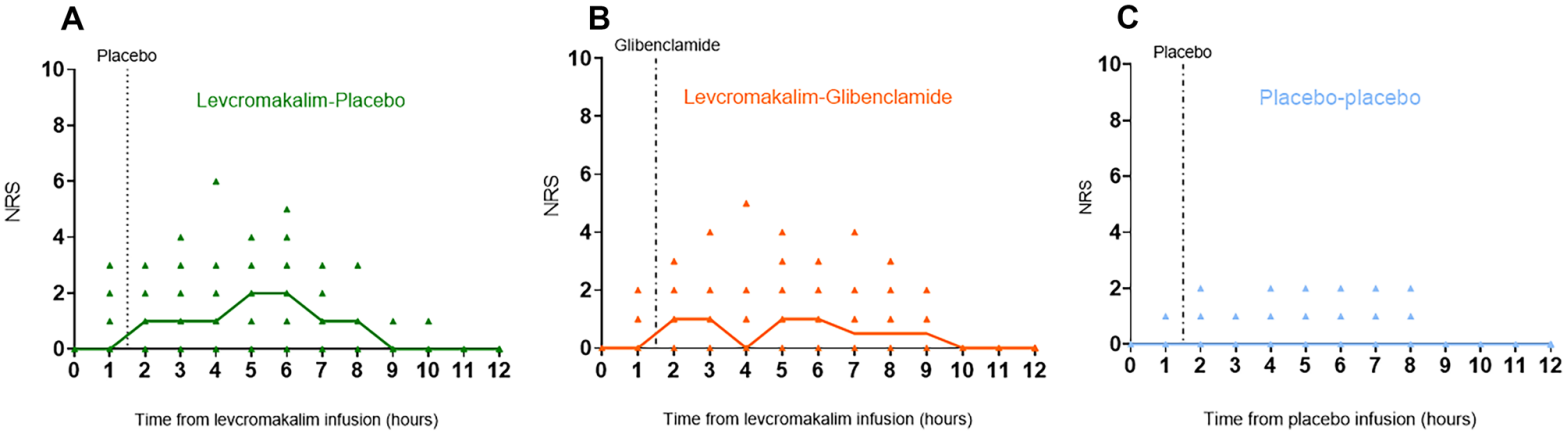
Headache intensity. Numerical rating scale (NRS) scores for headache intensity after infusion of levcromakalim or placebo and administration of oral glibenclamide or placebo, from baseline to 12 h. Dots represent individual headache scores of each participant on each study day. Thick line represents the median headache on **A** levcromakalim-placebo day, **B** levcromakalim-glibenclamide day, and **C** placebo-placebo day. The area under the curve (AUC) for headache intensity did not differ between the levcromakalim-placebo (494 ± 336.6) and the levcromakalim-glibenclamide day (417 ± 371.6) (*P* > 0.05). The AUC for headache intensity was significantly larger in the levcromakalim-placebo day (494 ± 336.6) compared to the placebo-placebo day (106.3 ± 215.8) (95% mean difference CI: 194.4–580.9) (*P* < 0.01)

Headache incidence was higher on levcromakalim-placebo day (15/15, 100%) and levcromakalim-glibenclamide day (13/15, 86%) compared to placebo-placebo day (7/15, 46%) (*P* = *0.013* and *P* = *0.041*, respectively). There was no difference in the headache incidence between levcromakalim-placebo day and levcromakalim-glibenclamide day (*P* = *0.479*). Median headache duration was 340 min (range 60–510) on levcromakalim-placebo day, 320 min (range 0–530) on levcromakalim-glibenclamide day, and 0 min (range 0–440) on placebo-placebo day. Median peak headache intensity was 2 (range 1–6) on levcromakalim-placebo day, 2 (range 0–5) on levcromakalim-glibenclamide day, and 0 (range 0–3) on placebo-placebo day (Table 1). We found no difference in the AUC_0–12 h_ for headache intensity between the levcromakalim-placebo (494 ± 336.6) day and the levcromakalim-glibenclamide day (417 ± 371.6) (*P* = *0.836*). The AUC_0–12 h_ for headache intensity was significantly larger in levcromakalim days compared to placebo-placebo day (106.3 ± 215.8) (*P* < *0.01*) (Fig. 4).

**Table 1:** Clinical characteristics of headache and associated symptoms in healthy volunteers after (0–12-h observation period)

**Participants**	**Peak headache** **(Duration of headache)**	**Headache characteristics**^**a**^	**Associated symptoms**^**b**^	**Migraine-like attacks**^**c**^	**Treatment (time)/efficacy**^**d**^
**1 W**					
Levcromakalim-placebo	360 min (340 m)	Diffuse & bilat/4/throb/ +	-/ + / +	Yes	-
Levcromakalim-glibenclamide	None	-	+/-/-	No	-
Placebo-placebo	None	-	-/+/-	No	-
**2 M**					
Levcromakalim-placebo	100 min (80 m)	Bilat/1/pres/-	-/-/-	No	No
Levcromakalim-glibenclamide	360 min (60 min)	Bilat/1/pres/-	-/-/-	No	-
Placebo-placebo	None	-	-/-/-	No	-
**3 W**					
Levcromakalim-placebo	360 min (370 m)	Bilat & Unilat/5/pres /-	+ /-/-	Yes	1000 mg paracetamol(6 h)/Yes
Levcromakalim-glibenclamide	140 min (450 min	Bilat & diffuse/3/pres/ +	-/-/-	No	No
Placebo-placebo	140 min (20 min)	Bilat/1/pres/ +	-/-/-	No	No
**4 M**					
Levcromakalim-placebo	140 min (440 m)	Bilat/3/pres/-	-/-/-	No	No
Levcromakalim-glibenclamide	40 min (30 min)	Bilat/1/pres/-	-/-/-	No	No
Placebo-placebo	None	-	-/-/-	No	No
**5 M**					
Levcromakalim-placebo	300 min (480)	Bilat/3/pres /-	+ /-/-	No	1000 mg paracetamol + 400 mg ibuprofen (6 h)/Yes
Levcromakalim-glibenclamide	220 min (530 min)	Bilat/3/pres/-	+ /-/-	No	Primperan 10 mg iv
Placebo-placebo	80 min (420 min)	Bilat/2/pres/-	+ /-/-	No	1000 mg paracetamol (5 h)/No 400 mg ibuprofen (8 h)/Yes
**6 W**					
Levcromakalim-placebo	420 min (260 m)	Bilat/3/pres/ +	-/-/ +	No	400 mg ibuprofen(8 h)/yes
Levcromakalim-glibenclamide	420 min (320 min)	Bilat/4/pres/ +	-/-/-	No	acetylsalicylic acid 150 mg (5 h)/No acetylsalicylic acid 150 mg (7 h)/Yes
Placebo-placebo	220 min (100 min)	Bilat/2/pres/-	-/-/-	No	No
**7 M**					
Levcromakalim-placebo	140 min (280 min)	Bilat/2/pres/-	-/-/-	No	No
Levcromakalim-glibenclamide	300 min (180 min)	Bilat/4/pres/-	-/-/-	No	No
Placebo-placebo	None	-	-/-/-	No	No
**8 M**					
Levcromakalim-placebo	300 min (160 min)	Unil/2/pres/ +	-/-/-	No	No
Levcromakalim-glibenclamide	300 min (300 min)	Bilat/2/pres & throb/ +	-/-/-	No	No
Placebo-placebo	70 min (230 min)	Bilat/1/pres/ +	-/-/-	No	No
**9 W**					
Levcromakalim-placebo	30 min (510 min)	Bilat/1/pres/ +	-/-/-	No	No
Levcromakalim-glibenclamide	300 min (530 min)	Bilat & diffuse/3/pres/-	+ /-/-	No	No
Placebo-placebo	50 min (440 min)	Bilat & diffuse/3/pres/ +	-/-/-	No	No
**10 W**					
Levcromakalim-placebo	240 min (450 min)	Bilat/6/pres/-	+ / + / +	Yes	1000 mg paracetamol + 400 mg ibuprofen (5 h)/Yes
Levcromakalim-glibenclamide	20 min (180 min)	Bilat/2/pres/-	-/-/-	No	No
Placebo-placebo	240 min (10 min)	Bilat/2/other/-	-/-/-	No	No
**11 M**					
Levcromakalim-placebo	300 min (60 min)	Diffuse/1/other (heavy head)/-	-/-/-	No	No
Levcromakalim-glibenclamide	None	-	-/-/-	No	No
Placebo-placebo	None	-	-/-/-	No	
**12 W**					
Levcromakalim-placebo	100 min (80 min)	Diffuse/1/pres/ +	-/-/-	No	No
Levcromakalim-glibenclamide	90 min (330 min)	Bilat& diffuse/1/pres/-	-/-/-	No	No
Placebo-placebo	None	-	-/-/-	No	No
**13 W**					
Levcromakalim-placebo	300 min (460 min)	Bilat & diffuse/4/throb/ +	-/-/-	No	1000 mg paracetamol & 400 mg ibuprofen (4,5 h)/Yes
Levcromakalim-glibenclamide	240 min (460 min)	Bilat & diffuse/5/pres &throb/ +	-/-/-	No	No
Placebo-placebo	None	-	-/-/-	No	No
**14 W**					
Levcromakalim-placebo	60 min (390 min)	Bilat/2/pres & throb/ -	+/-/-	Yes	No
Levcromakalim-glibenclamide	30 min (330 min)	Bilat/1/throb/ +	-/-/-	No	No
Placebo-placebo	20 min (140 min)	Bilat/1/press/ -	-/-/-	No	No
**15 W**					
Levcromakalim-placebo	220 min (320 min)	Diffuse/2/pres/ +	-/-/-	No	No
Levcromakalim-glibenclamide	120 min (360 min)	Bilat & diffuse/2/pres/ +	-/-/-	No	No
Placebo-placebo	None	-	-/-/-	No	No

*min* minutes, *W* woman, *M* man

^a^Localization/intensity/quality (throb = throbbing; pres = pressing; diffuse)/aggravation (by cough during in-hospital phase and by movement during out-hospital phase)

^b^Nausea/photophobia/phonophobia

^c^Migraine-like attacks must fulfil criteria B and C for migraine attack without aura according to ICHD-3 or mimic the patient’s usual migraine attacks and are treated with a rescue medication

^d^Pain freedom or pain relief (≥ 50% decrease of intensity) within 2 h

On levcromakalim-placebo day, 4/15 (26.6%) participants reported nausea, 2/15 photophobia (13.3%), and 3/15 phonophobia (20%) compared to 3/15 nausea (20%) and no photophobia and phonophobia on levcromakalim-glibenclamide day. On placebo-placebo day, 1/15 (6%) participant reported nausea, 1/15 (6%) photophobia, and none phonophobia. Four participants reported migraine-like headache [13] on levcromakalim-placebo day and none on the other two study days (Table 1). Five (33%) participants used rescue medication on levcromakalim-placebo day compared to 2 (13.3%) on levcromakalim-glibenclamide day (*P* > *0.05*) and one (6%) on placebo-placebo day (*P* > *0.05*).

### Vital Signs

**Fig. 5:**
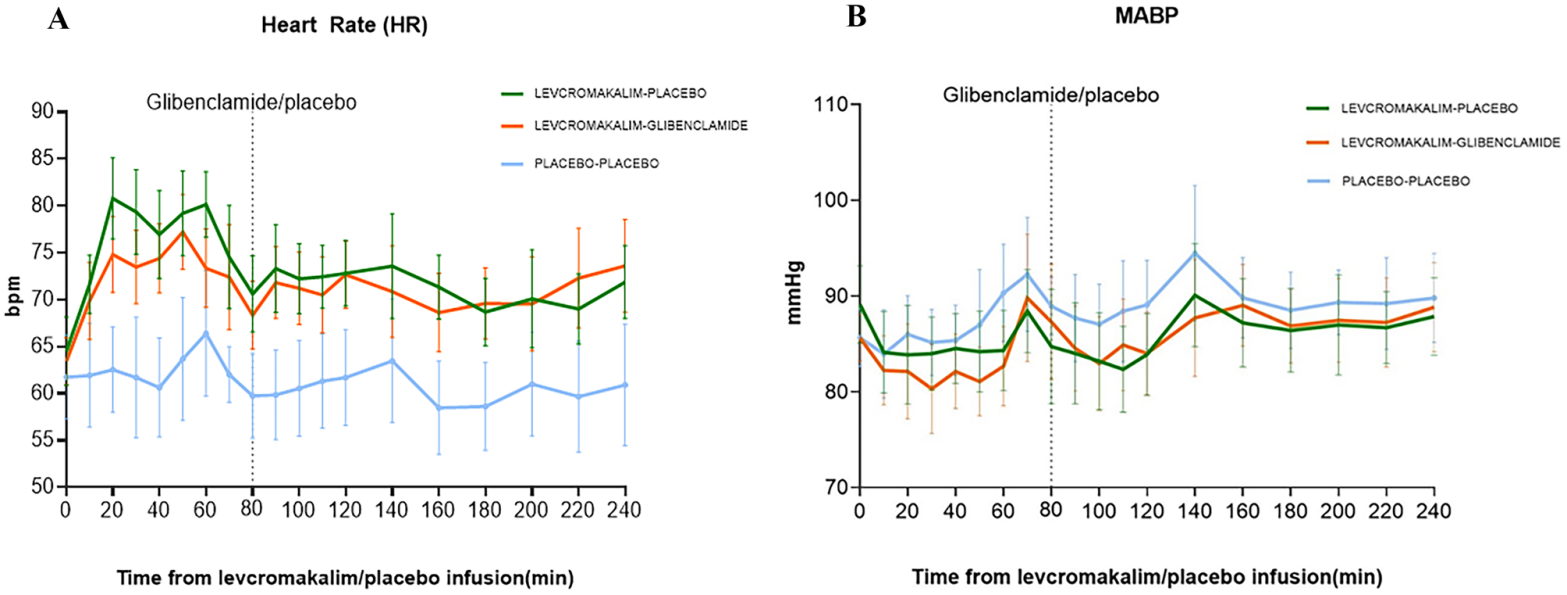
Hemodynamic parameters. **A** Mean change in heart rate (HR). Heart rate after infusion of levcromakalim/placebo and administration of oral glibenclamide/placebo, from baseline to T240 min. There was no difference in the AUC_0-240 min_ for HR on the levcromakalim-placebo day (17512 ± 1282) compared to levcromakalim-glibenclamide days (17148 ± 1477) (*P* > *0.05*). The AUC_0-240 min_ for HR was significantly larger in levcromakalim-placebo day and levcromakalim-glibenclamide day compared to the placebo-placebo day (14017 ± 2136) (*P* < *0.001)*. **B** Mean arterial blood pressure (MABP) after infusion of levcromakalim/placebo and administration of oral glibenclamide/placebo, from baseline to T240 min. Mean change in mean arterial blood pressure. There was no difference in the AUC_0-240 min_ for MABP between the three study days (levcromakalim-placebo: 20601 ± 2136, levcromakalim-glibenclamide: 20556 ± 1711, placebo-placebo: 20593 ± 1960) (*P* > *0.05*)

The AUC_0–240 min_ for HR was significantly larger on the levcromakalim-placebo (17512 ± 1282) and levcromakalim-glibenclamide days (17148 ± 1477) compared to placebo-placebo day (14017 ± 2136) (*P* < *0.001*) (Fig. 5A). There was no difference between levcromakalim-placebo and the levcromakalim-glibenclamide day (*P* = *0.346*). The AUC_0–240 min_ for MABP did not differ significantly between the three study days (levcromakalim-placebo: 20601 ± 2136, levcromakalim-glibenclamide: 20556 ± 1711, placebo-placebo: 20593 ± 1960, (*P* > *0.05 for all comparisons*)) (Fig. 5B).

## Discussion

The present study investigated the effect of glibenclamide treatment on levcromakalim-induced headache. The main findings are that levcromakalim induced headache in almost all healthy participants, which is consistent with results from previous studies [5], and glibenclamide posttreatment did not affect the headache incidence or changes in HR and MABP induced by levcromakalim. Glibenclamide was administered 1 h after levcromakalim infusion and its maximum plasma concentration (T_max_) is achieved 2–3 h following oral ingestion [14]. At 2.5 h post-glibenclamide administration, median headache score was 0 NRS on levcromakalim-glibenclamide day compared to 1 NRS on levcromakalim-placebo day. Although not significant, the observed headache pain relief on levcromakalim-glibenclamide day might be explained by glibenclamide T_max_. Interestingly, we have previously shown that glibenclamide pre-treatment delayed levcromakalim-induced headache by 2.5 h in healthy volunteers [15]. Calcitonin gene-related peptide (CGRP) and pituitary adenylate cyclase-activating polypeptide-38 (PACAP38) are potent migraine and headache triggering molecules [16–19]. The intracellular mechanisms underlying experimentally induced headache and migraine remain unclear. Binding of these peptides to their G protein-coupled receptors (GPCRs) in VSMCs activates complex intracellular cascades, including upregulation of cyclic adenosine monophosphate (cAMP), activation of protein kinase A (PKA), and eventually phosphorylation of several downstream molecules such as K_ATP_ channels [20]. In a preclinical model of migraine, glibenclamide attenuated cephalic hypersensitivity in spontaneous trigeminal allodynic rats [11], and glibenclamide almost completely inhibited cephalic hypersensitivity and pain responses following sensitization with CGRP, PACAP-38, or levcromakalim [10, 21]. In contrast, glibenclamide had no effect on the induced headache following infusion of CGRP, PACAP38, or levcromakalim in healthy participants [22, 23]. The above-mentioned studies were conducted in healthy participants. Given that administration of levcromakalim induces headache in healthy participants and migraine in migraine patients, it would be of great interest investigating the effect of glibenclamide in levcromakalim-induced migraine.

In preclinical studies, where glibenclamide blocked levcromakalim-induced trigeminal pain, it was administered intraperitoneally [10, 11, 21]. Parenteral formulations of glibenclamide are unavailable for clinical use. Glibenclamide is readily absorbed by the gastrointestinal tract with high bioavailability following oral administration [24]. The dose of glibenclamide (10 mgs oral ingestion) used in our study is the maximum tolerated dose in humans to minimize the risk of severe hypoglycemia. Given the higher metabolic rate of rodents, the glibenclamide dose (1 mg/kg) used in preclinical settings should be translated into the human equivalent dose ((HED) = animal dose × animal_km_/human_km_) [25]. Km factors of mouse and rat are 3 and 6, respectively, whereas km factor for an adult human is 37. Thus, the equivalent dose in humans is 0.08–0.16 mg/kg. In the present study, 0.15 mg/kg of glibenclamide was administered (mean weight of participants: 65.2 kg). Thus, the observed conflicting results on the efficacy of comparable doses of glibenclamide suggest significant interspecies differences and highlight the possibility of different expression of K_ATP_ channel subunits among human and rodents.

K_ATP_ channels consist of four pore-forming K^+^ inwardly rectifying (Kir) subunits and four regulatory sulfonylurea receptor subunits (SUR) [26, 27]. Distinct combinations of the Kir and SUR subunits determine structure and function of different subtypes of K_ATP_ channels. Levcromakalim activates more potently the Kir6.1/SUR2B channels expressed in VSMCs and also in TG and TNC [28, 29]. Whether levcromakalim can cross the blood–brain barrier (BBB) is uncertain. Based on its small molecular weight (286.33 Da) and lipophilic properties, we could not completely rule out a direct action of levcromakalim on neuronal K_ATP_ channels [30]. Activation of K_ATP_ channels expressed in neurons leads to hyperpolarization and potassium efflux [31]. The increase in extracellular potassium might subsequently activate hyperpolarization-activated cyclic nucleotide-gated (HCN) channels expressed in the trigeminal ganglion and the CNS [32]. HCN channels have been implicated in inflammatory and neuropathic pain, and their role in migraine pathophysiology has not yet been elucidated [33, 34]. Of note, glibenclamide is a non-selective K_ATP_ channel blocker with a higher affinity for the SUR1 subunit, compared to other SUR subunits [35, 36]. To date, no selective K_ATP_ channel antagonists are available. It is crucial for our understanding of the involvement of K_ATP_ channels in migraine headache that future studies examine the selective blockade of K_ATP_ channel subunits.

## Conclusion

We have presented the first study using non-specific K_ATP_ channel blocker glibenclamide as posttreatment to levcromakalim induced headache in healthy participants. Glibenclamide did not inhibit the headache induced by levcromakalim. Selective K_ATP_ channel blockers are needed to investigate the involvement of the different K_ATP_ channel subunits in migraine pathophysiology.

## Supplementary Information

Below is the link to the electronic supplementary material.

Supplementary file1 (PDF 495 KB)

Supplementary file2 (PDF 5268 KB)

Supplementary file3 (PDF 451 KB)

Supplementary file4 (PDF 6295 KB)

## Data Availability

Data are available from the authors upon reasonable request and with permission of data protection department of Rigshospitalet, Copenhagen, Denmark.

## References

[1] Migraine AM (2020). Ropper AH, editor. N Engl J Med.

[2] Kokoti L, Al-Karagholi MAM, Ashina M. Latest insights into the pathophysiology of migraine: the ATP-sensitive potassium channels. Curr Pain Headache Rep. 2020;24(12). 10.1007/s11916-020-00911-6 33270149

[3] Ploug KB, Sørensen MA, Strøbech L, Klaerke DA, Hay-Schmidt A, Sheykhzade M (2008). KATP channels in pig and human intracranial arteries. Eur J Pharmacol.

[4] Ploug KB, Baun M, Hay-Schmidt A, Olesen J, Jansen-Olesen I (2010). Presence and vascular pharmacology of KATP channel subtypes in rat central and peripheral tissues. Eur J Pharmacol.

[5] Al-Karagholi MAM, Ghanizada H, Hansen JM, Skovgaard LT, Olesen J, Larsson HBW (2019). Levcromakalim, an adenosine triphosphate-sensitive potassium channel opener, dilates extracerebral but not cerebral arteries. Headache.

[6] Al-Karagholi MAM, Hansen JM, Guo S, Olesen J, Ashina M (2019). Opening of ATP-sensitive potassium channels causes migraine attacks: a new target for the treatment of migraine. Brain.

[7] Kitazono T, Heistad DD, Faraci FM. Role of ATP-sensitive K+ channels in CGRP-induced dilatation of basilar artery in vivo. Am J Physiol - Hear Circ Physiol. 1993;265(2 34–2):581–5. 10.1152/ajpheart.1993.265.2.H581 8368361

[8] Quayle JM, Bonev AD, Brayden JE, Nelson MT (1994). Calcitonin gene-related peptide activated ATP-sensitive K+ currents in rabbit arterial smooth muscle via protein kinase A. J Physiol.

[9] Faraci FM, Brian JE (1994). Nitric oxide and the cerebral circulation. Stroke.

[10] Christensen SL, Rasmussen RH, Ernstsen C, La CS, David A, Chaker J (2021). CGRP-dependent signalling pathways involved in mouse models of GTN-cilostazol-and levcromakalim-induced migraine. Cephalalgia.

[11] Christensen SL, Munro G, Petersen S, Shabir A, Jansen-Olesen I, Kristensen DM (2020). ATP sensitive potassium (KATP) channel inhibition: a promising new drug target for migraine. Cephalalgia.

[12] Vincent M, Wang S. Headache Classification Committee of the International Headache Society (IHS) The International Classification of Headache Disorders, 3rd edition. Cephalalgia. 2018;38(1):1–211. 10.1177/0333102417738202 29368949

[13] Ashina M, Terwindt GM, Al-Karagholi MAM, de Boer I, Lee MJ, Hay DL (2021). Migraine: disease characterisation, biomarkers, and precision medicine. Lancet.

[14] Coppack S, Lant A, McIntosh C, Rodgers A (1990). Pharmacokinetic and pharmacodynamic studies of glibenclamide in non-insulin dependent diabetes mellitus. Br J Clin Pharmacol.

[15] Al-Karagholi MA-M, Ghanizada H, Kokoti L, Paulsen JS, Hansen JM, Ashina M. Effect of K<inf>ATP</inf> channel blocker glibenclamide on levcromakalim-induced headache. Cephalalgia. 2020;40(10). 10.1177/0333102420949863 32806954

[16] Lassen LH, Haderslev PA, Jacobsen VB, Iversen HK, Sperling B, Olesen J (2002). CGRP may play a causative role in migraine. Cephalalgia.

[17] Falkenberg K, Rønde Bjerg H, Yamani N, Olesen J (2020). Sumatriptan does not antagonize CGRP-induced symptoms in healthy volunteers. Headache.

[18] Schytz HW, Birk S, Wienecke T, Kruuse C, Olesen J, Ashina M (2009). PACAP38 induces migraine-like attacks in patients with migraine without aura. Brain.

[19] Amin FM, Asghar MS, Guo S, Hougaard A, Hansen AE, Schytz HW (2012). Headache and prolonged dilatation of the middle meningeal artery by PACAP38 in healthy volunteers. Cephalalgia.

[20] Al-Karagholi MAM, Hansen JM, Severinsen J, Jansen-Olesen I, Ashina M (2017). The KATP channel in migraine pathophysiology: a novel therapeutic target for migraine. J Headache Pain.

[21] Ernstsen C, Christensen SL, Rasmussen RH, Nielsen BS, Olesen J, Kristensen DM (2022). The PACAP pathway is independent of CGRP in mouse models of migraine: possible new drug target?. Brain.

[22] Coskun H, Elbahi FA, Al-Karagholi MA, Ghanizada H, Sheykhzade M, Ashina M (2021). The effect of K _ATP_ channel blocker glibenclamide on CGRP-induced headache and hemodynamic in healthy volunteers. Front Physiol.

[23] Kokoti L, Al-Mahdi Al-Karagholi M, Elbahi FA, Coskun H, Ghanizada H, Amin FM (2022). Effect of K ATP channel blocker glibenclamide on PACAP38-induced headache and hemodynamic. Cephalalgia.

[24] Lev JD, Zeidler A, Kumar D (1987). Glyburide and glipizide in treatment of diabetic patients with secondary failures to tolazamide or chlorpropamide. Diabetes Care.

[25] Reagan-shaw S, Nihal M, Ahmad N (2008). Dose translation from animal to human studies revisited. FASEB J.

[26] Clement JP, Kunjilwar K, Gonzalez G, Schwanstecher M, Panten U, Aguilar-Bryan L (1997). Association and stoichiometry of K(ATP) channel subunits. Neuron.

[27] Kuang Q, Purhonen P, Hebert H (2015). Structure of potassium channels. Cell Mol Life Sci.

[28] Rodrigo G, Standen N (2005). ATP-sensitive potassium channels. Curr Pharm Des.

[29] Ploug KB, Boni LJ, Baun M, Hay-Schmidt A, Olesen J, Jansen-Olesen I (2008). K ATP channel expression and pharmacological in vivo and in vitro studies of the K ATP channel blocker PNU-37883A in rat middle meningeal arteries. Br J Pharmacol.

[30] Levcromakalim | C16H18N2O3 - PubChem. Available from: https://pubchem.ncbi.nlm.nih.gov/compound/Levcromakalim.

[31] Inagaki N, Seino S. ATP-sensitive potassium channels: structures, functions, and pathophysiology. Japanese J Physiol. 1998;48:397–412. 10.2170/jjphysiol.48.397 10021494

[32] Emery EC, Young GT, Berrocoso EM, Chen L, McNaughton PA (2011). HCN2 ion channels play a central role in inflammatory and neuropathic pain. Science.

[33] Haanes KA, Edvinsson L (2019). Pathophysiological mechanisms in migraine and the identification of new therapeutic targets. CNS Drugs.

[34] Takasu K, Ono H, Tanabe M (2010). Spinal hyperpolarization-activated cyclic nucleotide-gated cation channels at primary afferent terminals contribute to chronic pain. Pain.

[35] Rubaiy HN (2016). The therapeutic agents that target ATP-sensitive potassium channels. Acta Pharm.

[36] Al-Karagholi MA-M, Sode M, Gozalov A, Ashina M. The vascular effect of glibenclamide: a systematic review. Cephalalgia Rep. 2019;2.

